# A non-human primate in vitro functional assay for the early evaluation of TB vaccine candidates

**DOI:** 10.1038/s41541-020-00263-7

**Published:** 2021-01-04

**Authors:** Rachel Tanner, Andrew D. White, Charelle Boot, Claudia C. Sombroek, Matthew K. O’Shea, Daniel Wright, Emily Hoogkamer, Julia Bitencourt, Stephanie A. Harris, Charlotte Sarfas, Rachel Wittenberg, Iman Satti, Helen A. Fletcher, Frank A. W. Verreck, Sally A. Sharpe, Helen McShane

**Affiliations:** 1 The Jenner Institute, University of Oxford, Oxford, UK; 2 Public Health England, Salisbury, UK; 3 TB Research Group, Department of Parasitology, Biomedical Primate Research Centre, Rijswijk, Netherlands; 4 Institute of Immunology and Immunotherapy, College of Medical and Dental Sciences, University of Birmingham, Birmingham, United Kingdom; 5 Gonҫalo Moniz Institute, Oswaldo Cruz Foundation (FIOCRUZ), Salvador, Brazil; 6 London School of Hygiene and Tropical Medicine, London, UK

**Keywords:** Immunology, Microbiology, Biomarkers, Medical research, Diseases

## Abstract

We present a non-human primate mycobacterial growth inhibition assay (MGIA) using in vitro blood or cell co-culture with the aim of refining and expediting early tuberculosis vaccine testing. We have taken steps to optimise the assay using cryopreserved peripheral blood mononuclear cells, transfer it to end-user institutes, and assess technical and biological validity. Increasing cell concentration or mycobacterial input and co-culturing in static 48-well plates compared with rotating tubes improved intra-assay repeatability and sensitivity. Standardisation and harmonisation efforts resulted in high consistency agreements, with repeatability and intermediate precision <10% coefficient of variation (CV) and inter-site reproducibility <20% CV; although some systematic differences were observed. As proof-of-concept, we demonstrated ability to detect a BCG vaccine-induced improvement in growth inhibition in macaque samples, and a correlation between MGIA outcome and measures of protection from in vivo disease development following challenge with either intradermal BCG or aerosol/endobronchial *Mycobacterium tuberculosis* (*M.tb*) at a group and individual animal level.

## Introduction

Tuberculosis (TB) is the leading cause of death due to an infectious pathogen with an estimated 10 million new cases and 1.42 million deaths in 2019^1^. The only available vaccine, BCG, is insufficient and a new vaccine is urgently needed. A major barrier to the development of an efficacious vaccine is the lack of a validated correlate of protection from TB, although several potential biomarkers have been proposed^2–4^. As such, novel TB vaccine candidates or regimens are currently evaluated using preclinical models including non-human primates (NHPs). The use of NHPs in medical research is emotive^5^, and assessment of protective TB vaccine efficacy currently necessitates in vivo challenge with virulent *Mycobacterium tuberculosis* (*M.tb*), a procedure resulting in disease development and classified in macaques as ‘Moderate Severity’ under UK ASPA licensure and European legislation^6^. Sufficient animal numbers are required to ensure reliable conclusions, and experiments are relatively time- and cost-consuming requiring high biosafety containment facilities^7^. Macaques are widely considered the most relevant model for the evaluation of TB vaccine candidates, and are essential in supporting advancement into clinical trials^8^. It is critical that new tools are developed and exploited to facilitate vaccine testing in macaques in line with the 3Rs principles of replacement, reduction and refinement of the use of animals in scientific research^9^.

In the absence of a validated correlate or surrogate of protection from TB, functional assays such as mycobacterial growth inhibition assays (MGIAs) may offer a valuable complementary tool for vaccine evaluation. The ‘sum-of-the-parts’ outcome of these in vitro/ex vivo assays measures the contributions of multiple components of the immune response and their interactions within their immune environment. As such, they aim to give an unbiased measure of the ability of whole blood or cell samples to control mycobacterial growth without the limitation of having to pre-select individual immune parameters of unclear relevance^10^. A successful, validated MGIA could be used to down-select experimental TB vaccine candidates at an early stage of development, reducing the number progressing to virulent *M.tb* challenge experiments. Control of different mycobacterial strains and isolates, and underlying immune mechanisms, can be explored using cells from a single group of vaccinated animals, further reducing animal numbers. Several MGIAs have been reported using samples from humans^11–16^, mice^17–23^ and cattle^24–26^, many demonstrating ability to detect a BCG vaccine-induced improvement in growth inhibition. Furthermore, they have been applied to clinical cohorts of patients with latent TB infection and active TB disease to study immune mechanisms or correlates of mycobacterial control including monocytes, memory B-cells and cytokine responses^27,28^.

To date, there are no reports of an MGIA for use in the macaque model of TB vaccine development, although some of the authors have recently applied the assay described here to detect a signal after BCG vaccination and experimental *M.tb* infection in macaques^29,30^. A macaque MGIA would be particularly valuable not only from a 3Rs perspective, but also as an opportunity for biological assay validation by correlating MGIA outcome with direct measures of in vivo protection from *M.tb* challenge, which is not possible in humans. The assay could then be bridged to use in humans with increased confidence. In the absence of an effective TB vaccine, BCG vaccination, which offers partial and quantifiable protection against *M.tb* challenge in NHPs^31–35^, is a bench-mark for assessing potential correlates or surrogates of protection. As protection afforded by BCG vaccination in humans and NHPs is variable^36,37^, we have considered the association between outcomes of in vivo challenge and MGIA on a per-individual as well as group basis. Alongside our efforts to optimise and biologically validate the NHP MGIA, we sought to transfer and harmonise a standardised protocol between laboratories and assess reproducibility at multiple levels. Such activities are critical to ensure that 3Rs impact is maximised and comparable information can be extracted from ongoing and future studies of different preclinical vaccine candidates across organisations.

## Results

### Mycobacterial growth in the direct whole blood MGIA is inhibited following BCG vaccination and correlates with BCG recovered from the lymph node following in vivo BCG challenge

**Fig. 1:**
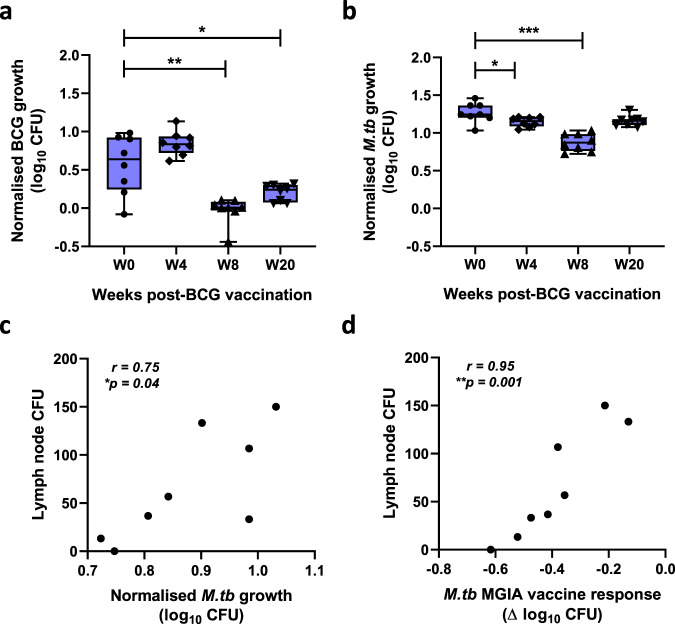
Mycobacterial growth inhibition in the direct whole blood MGIA is enhanced following BCG vaccination and correlates with BCG recovered from the lymph node following in vivo BCG challenge. Samples were collected from eight cynomolgus macaques from Study 1 that received BCG vaccination by the ID route. The MGIA was performed using whole blood collected at baseline, 4 weeks, 8 weeks and 20 weeks post-BCG vaccination using (**a**) BCG Pasteur or (**b**) *M.tb* H37Rv as the inoculum. At 20 weeks after vaccination, all animals were challenged by exposure to ID BCG, and CFU recovered from the lymph node was quantified 2 weeks later. Spearman’s rank correlations are shown between lymph node CFU and (**c**) *M.tb* growth at 8 weeks post-BCG vaccination or (**d**) *M.tb* MGIA vaccine response (post-vaccination growth–baseline growth) at 8 weeks post-BCG vaccination relative to baseline. Points represent individual animals with the mean of two co-culture replicates. Normalised mycobacterial growth is equal to (log_10_ CFU of sample – log_10_ CFU of inoculum control). Boxes indicate the median value with the interquartile range and whiskers indicate the minimum and maximum values. A repeated-measures one-way ANOVA with Dunnett’s multiple comparisons test was performed (**a**, **b**) where *p-value < 0.05, **p-value < 0.01, and ***p-value < 0.001.

Samples were taken from eight cynomolgus macaques from Study 1 that received BCG vaccination by the intradermal (ID) route. Applying the whole blood MGIA, significantly improved control of BCG growth was observed at 8 weeks and 20 weeks post-BCG vaccination compared with baseline (*p* = 0.0048, mean difference (MD) = 0.60 log_10_ CFU, 95% CI 0.23–0.97; and *p* = 0.0412, MD = 0.37 log_10_ CFU, 95% CI 0.02–0.72 respectively; RM one-way ANOVA with Dunnett’s post-test, *p* = 0.0002, *F*(1.44, 10.06) = 27.2; Fig. 1a). Using *M.tb* H37Rv as the MGIA inoculum, there was significantly reduced *M.tb* growth at 4 weeks and 8 weeks post-BCG vaccination compared with baseline (*p* = 0.040, MD = 0.12 log_10_ CFU, 95% CI 0.006–0.23; and *p* = 0.0006, MD = 0.39 log_10_ CFU, 95% CI 0.22–0.56 respectively; RM one-way ANOVA with Dunnett’s post-test, *p* < 0.0001, *F*(2.46, 17.18) = 27.40; Fig. 1b), but not at 20 weeks post-BCG vaccination. Using *M.tb* as the MGIA inoculum resulted in superior repeatability between replicate cultures compared with BCG (median CV = 2.3% and 3.9% respectively; *p* = 0.02, two-tailed Wilcoxon test; Supplementary Fig. 1a); the intra-class correlation coefficient (ICC) for *M.tb* was 0.69 (‘substantial agreement’) and for BCG was 0.56 (‘moderate agreement’). However, the MGIA vaccine response (post-vaccination growth – baseline growth) was greater at 8 weeks and 20 weeks post-vaccination using BCG compared with *M.tb*, although this was not statistically significant (Supplementary Fig. 1b).

All animals were challenged ID with high-dose BCG (as a potential surrogate for virulent *M.tb*) at 21 weeks post-BCG vaccination, and 2 weeks later a challenge site biopsy and axillary lymph nodes (LN) were taken and BCG quantified by solid agar culture and qPCR. As previously reported, there was no difference in biopsy CFU recovery between the naïve and BCG-vaccinated groups^38^, and we did not see an association between this measure and the MGIA response. However, there was a significant reduction in LN CFU recovery from the BCG-vaccinated group compared with the naïve group following BCG challenge^38^, and we observed a significant correlation between LN CFU and *M.tb* growth in the MGIA at peak of response (8 weeks post-vaccination) on a per-animal basis (*p* = 0.04, *r* = 0.75, Spearman’s, Fig. 1c). Furthermore, there was a significant association between LN CFU and *M.tb* MGIA vaccine response (post-vaccination growth – baseline growth) at 8 weeks post-BCG vaccination (*p* = 0.001, *r* = 0.95, Spearman’s, Fig. 1d). These associations were not statistically significant using the BCG MGIA, although there was a correlation between BCG and *M.tb* growth in the two assays (*p* = 0.0001, *r* = 0.63, Spearman’s, data not shown).

### Optimisation of the NHP direct PBMC MGIA: increasing cell number and co-culturing in static 48-well plates improves intra-assay repeatability and assay sensitivity

Due to emerging concerns regarding haemoglobin levels confounding the whole blood MGIA in small animal models^29^, batch effects associated with performing the assay in real-time^39,40^, and to maximise cryopreserved sample use from historical studies to reduce the number of animals used^41^, we sought to develop a cryopreserved PBMC-based MGIA for use with macaque samples. Samples were taken from 12 Rhesus macaques enrolled into Study 2; six unvaccinated controls, and six of which received BCG vaccination by the ID route^38^. The MGIA was performed using PBMC from baseline and 8 weeks post-BCG vaccination according to the previously described ‘in-tube protocol’ co-culturing 1 × 10^6^ cells with 10% pooled human serum and BCG in 2 ml screw-cap 360° rotating tubes^39^.

**Fig. 2:**
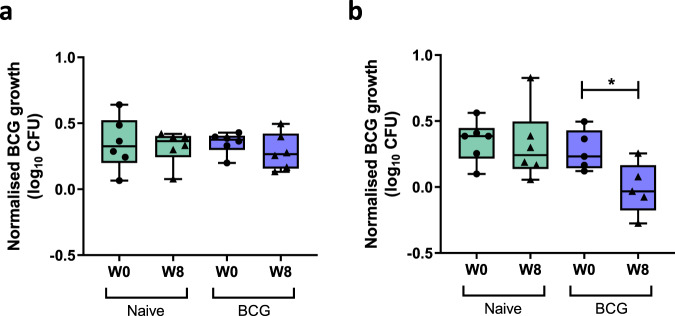
Optimisation of the NHP direct PBMC MGIA. Samples were collected from 12 Rhesus macaques from Study 2, six of which were naïve unvaccinated controls (green boxes), and six of which received BCG vaccination by the intradermal (ID) route (blue boxes). The MGIA was performed using PBMC from baseline (circles) and 8 weeks post-BCG vaccination (triangles) according to the original ‘in-tube protocol’ co-culturing in 2 ml screw-cap rotating tubes with (**a**) 1 × 10^6^ PBMC or (**b**) 5 × 10^6^ PBMC and a BCG inoculum of 500 CFU. Points represent individual animals with the mean of two co-culture replicates (**a**) or a single co-culture (**b**). Normalised mycobacterial growth is equal to (log_10_ CFU of sample – log_10_ CFU of inoculum control). Boxes indicate the median value with the interquartile range and whiskers indicate the minimum and maximum values. A one-way ANOVA with Sidak’s multiple comparisons test was performed where *p-value < 0.05.

Using a standard inoculum of 500 CFU BCG, a vaccine-induced improvement in growth inhibition was not detected (Fig. 2a). When the mycobacterial input was reduced to 50 CFU, intra-assay variability between replicate cultures increased (median CV for 500 and 50 CFU = 2.59 and 6.14% respectively) and again an effect of vaccination on mycobacterial growth was not detected (data not shown). We therefore reduced the multiplicity of infection (MOI) by increasing cell number rather than reducing inoculum, co-culturing 5 × 10^6^ cells with 500 CFU BCG in singlicate. Under these conditions, a modest reduction in MGIA growth was observed following BCG vaccination (*p* = 0.026, *t*(4) = 3.44, MD = −0.29 log_10_ CFU, 95% CI −0.52 to −0.06, paired *t*-test; one-way ANOVA with Sidak’s post-test, *p* = 0.038, *F*(3, 18) = 3.47; Fig. 2b). At 21 weeks, all Study 2 animals were challenged ID with a standard dose of BCG, and 2 weeks later a challenge site biopsy was taken and BCG quantified by culture on solid agar and qPCR. Axillary LNs were not collected in this study. As previously reported, the amount of BCG recovered from each biopsy was very low, and a difference in BCG recovery between the naïve and BCG-vaccinated groups was not detected^38^. We did not see an association between biopsy CFU and MGIA response using either assay protocol (data not shown).

It has recently been reported that co-culture of cells and mycobacteria in static tissue culture plates with CO_2_ improves cell viability and reproducibility in mouse splenocyte and human PBMC direct MGIAs^42,43^. We therefore compared the in-tube protocol with co-culture in 48-well plates using 1 × 10^6^ or 3 × 10^6^ cells collected from rhesus macaques from Study 3. Intra-assay repeatability between replicates, as measured by CV, did not differ between 1 × 10^6^ and 3 × 10^6^ cells for either condition, although repeatability was improved using plates compared with tubes, which was significant for the 1 × 10^6^ cells condition (*p* = 0.007, MD = 2.86%, 95% CI 0.62–5.10; one-way ANOVA with Tukey’s post-test, *p* = 0.0038, *F*(3, 73) = 4.87; Supplementary Fig. 2a). The ICC showed ‘slight to fair agreement’ between replicates using the in-tube protocol (ICC = 0.493 and 0.301 for 1 × 10^6^ and 3 × 10^6^ cells respectively); but ‘substantial agreement’ between replicates using the 48-well plate protocol (ICC = 0.795 and 0.605 for 1 × 10^6^ and 3 × 10^6^ cells respectively).

**Fig. 3:**
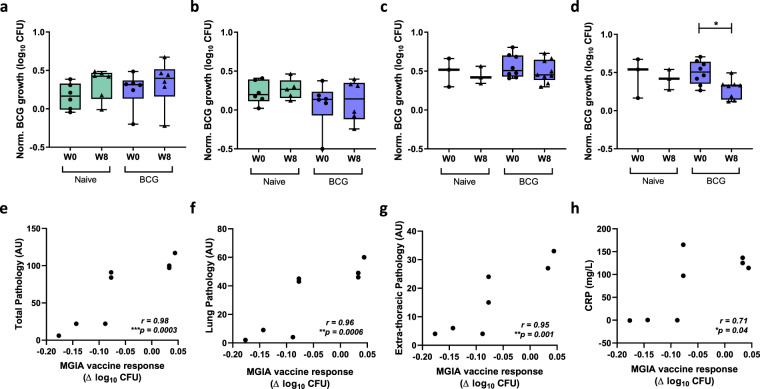
Optimisation of the NHP direct PBMC MGIA. Samples were collected from 24 Rhesus macaques from Study 3, nine of which were naïve unvaccinated controls (green boxes), and 15 of which received BCG vaccination by the intradermal (ID) route (blue boxes). The MGIA was performed using PBMC from baseline (circles) and 8 weeks post-BCG vaccination (triangles) according to the original ‘in-tube protocol’ co-culturing in 2 ml screw-cap rotating tubes (**a**, **b**) or in static 48-well tissue culture plates (**c**, **d**). Co-cultures contained either 1 × 10^6^ PBMC (**a**, **c**) or 3 × 10^6^ PBMC (**b**, **d**). Points represent individual animals with the mean of two co-culture replicates. Normalised mycobacterial growth is equal to (log_10_ CFU of sample – log_10_ CFU of inoculum control). Boxes indicate the median value with the interquartile range and whiskers indicate the minimum and maximum values. A one-way ANOVA was performed with Sidak’s multiple comparisons test where *p-value < 0.05. At 38 weeks after BCG vaccination, all animals were challenged by endobronchial installation of *M.tb* and measures of pathology were determined 2 weeks later. Correlations are shown between the MGIA vaccine response (post-vaccination growth – baseline growth) and (**e)** total pathology, (**f**) lung pathology, (**g**) extra-thoracic pathology and (**h**) CRP using Spearman’s rank correlation coefficient.

Due to limited cell availability, paired samples from Study 3 were randomised to either the in-tube protocol with 1 × 10^6^ and 3 × 10^6^ cells (*n* = 6 naïve and *n* = 6 BCG vaccinated animals), or in a separate experiment, the 48-well plate protocol with 1 × 10^6^ and 3 × 10^6^ cells (*n* = 3 naïve and *n* = 9 BCG vaccinated animals). The randomised groups showed similar levels of in vivo protection by all primary outcomes (data not shown). A vaccine-induced improvement in growth inhibition was not detected using the in-tube protocol for either cell input (Fig. 3a, b). However, applying the 48-well plate protocol, there was a trend towards improved mycobacterial growth inhibition following BCG vaccination using 1 × 10^6^ cells (Fig. 3c) and a significant improvement using 3 × 10^6^ cells (*p* = 0.018, *t*(6) = 3.24, MD = −0.25 log_10_ CFU, 95% CI −0.43 to −0.06, paired *t*-test; *p* = 0.029, MD = 0.22 log_10_ CFU, 95% 0.02–0.41, one-way ANOVA with Sidak’s post-test, *p* = 0.086, *F*(3, 18) = 2.58; Fig. 3d). The MGIA vaccine response (post-vaccination growth – baseline growth) was greater using 3 × 10^6^ compared with 1 × 10^6^ cells (*p* = 0.013, *t*(7) = 3.30, MD = −0.20 log_10_ CFU, 95% CI −0.34 to −0.06, paired *t*-test; Supplementary Fig. 2b), and there was a significant correlation between MGIA outcome for the two conditions (*p* = 0.02, *r* = 0.50, Spearman’s; Supplementary Fig. 2c).

All animals in Study 3 were challenged in vivo with *M.tb* Erdman by endobronchial installation at 38 weeks post-BCG vaccination. The clinical parameters measured at necropsy were time to humane endpoint (if fixed endpoint not reached), total pathology, lung pathology, extra-thoracic pathology, lung CFU, C-reactive protein (CRP) and weight change. Absolute growth in the MGIA at week 8 was not associated with any of the measures of protection, but MGIA vaccine response (post-vaccination growth – baseline growth) using 1 × 10^6^ cells correlated with total pathology, lung pathology, extra-thoracic pathology and CRP on a per-animal basis (*p* = 0.0003, *r* = 0.98; *p* = 0.0006, *r* = 0.96; *p* = 0.001, *r* = 0.95; and *p* = 0.048, *r* = 0.71 respectively, Spearman’s, Fig. 3e–h).

### Standardisation and harmonisation of the NHP direct PBMC MGIA results in consistency agreement across replicates, assay runs and sites

Based on the findings of our optimisation experiments, the limitations of cell availability, and to ensure consistency with the equivalent human assay^43^, the conditions of 3 × 10^6^ cells co-cultured in 48-well plates with 500 CFU BCG were selected for future experiments. Side-by-side operator training was conducted to transfer the optimised protocol from the developer institute (henceforth referred to as site 1) to two additional end-user institutes (sites 2 and 3).

PBMC samples collected from macaques enrolled into studies described in this paper were assayed in duplicate to assess intra-assay repeatability at the three different sites. The median CV between replicate co-cultures was 2.69% (range 0.59–6.12%, *n* = 8), 1.67% (range 0.78–8.52%, *n* = 5) and 2.71% (range 0–7.33%, *n* = 5) at sites 1, 2 and 3 respectively. The ICC values were 0.90 (‘almost perfect’ agreement), 0.34 (‘fair’ agreement) and 0.95 (‘almost perfect’ agreement) respectively (Table 1).

**Table 1:** NHP MGIA intra-assay repeatability.

	Site 1 (*n* = 11)	Site 2 (*n* = 4)	Site 3 (*n* = 13)
CV (%)	2.69	1.67	2.71
ICC	0.90	0.34	0.95

The median CV and ICC between replicate co-cultures was measured at sites 1, 2 and 3.

**Fig. 4:**
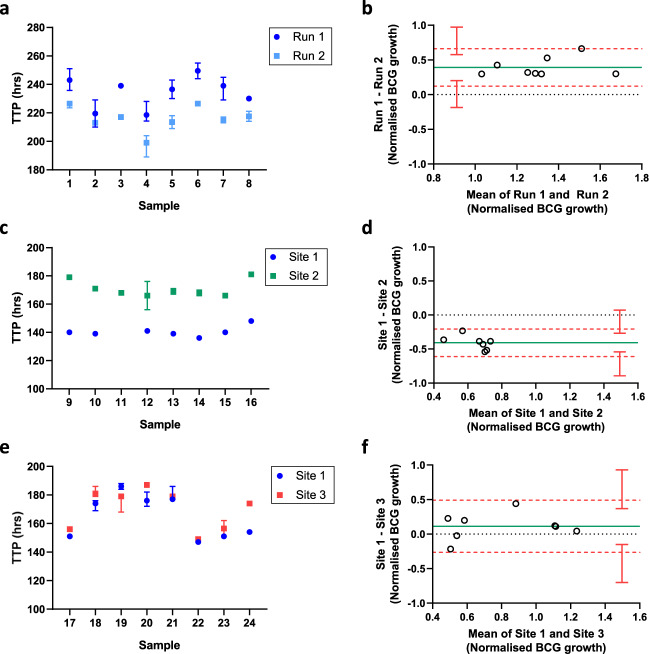
Standardisation and harmonisation of the NHP direct PBMC MGIA. The MGIA (3 × 10^6^ cells, 500 CFU BCG in static 48-well plates) was performed using PBMC collected from various studies described in this paper. A single sample set (*n* = 8) was assayed on two separate occasions at site 1 to assess inter-assay precision (**a**, **b**). Inter-site comparisons were conducted between sites 1 (blue) and 2 (green) (**c**, **d**) and between sites 1 (blue) and 3 (red) (**e**, **f**) using different shared sample sets (*n* = 8). Cultures were performed in duplicate or triplicate where cell availability allowed; data are expressed as median values with interquartile range (**a**, **c**, **e**). TTP = BACTEC MGIT time to positivity. **b**, **d**, **f** show Bland-Altman plots where the solid green line indicates the mean difference between measurements and the dotted red line indicates the upper and lower limits of agreement (mean difference ±1.96 standard deviation of the difference) with red vertical bars showing the 95% confidence intervals for the limits of agreement. Normalised mycobacterial growth is equal to (log_10_ CFU of sample – log_10_ CFU of inoculum control).

A single sample set (*n* = 8) was assayed on two separate occasions at site 1 to assess inter-assay precision (Fig. 4a). The median CV between assay runs was 6.83% (range 2.13 to 7.76%) with an ICC value of 0.80 (‘substantial’ agreement). While there was a strong consistency agreement, mycobacterial growth was systematically higher (shorter TTP) in run 2. As shown by Bland-Altman analysis relating the difference between paired measurements to the mean of the pair, this bias was not fully compensated by normalising growth using the direct-to-MGIT inoculum controls (mean bias = 0.39 log_10_ CFU, Fig. 4b). However, the difference between the highest and lowest responses was consistent between runs (0.66 and 0.64 log_10_ CFU respectively), and all samples were within 95% limits of agreement (the interval of 1.96 standard deviations of the measurement differences either side of the mean difference), which extended from 0.12 (95% CI, −0.19 to 0.21) to 0.66 (95% CI, 0.58–0.97) log_10_ CFU.

Finally, inter-site comparisons were conducted between sites 1 and 2 (Fig. 4c) and sites 1 and 3 (Fig. 4e) using a shared BCG Pasteur Aeras stock and two different shared sample sets (*n* = 8). Between sites 1 and 2, the median CV was 14.19% (range 11.57–17.29%) with an ICC value of 0.57 (‘moderate’ agreement; Table 2). Mycobacterial growth was consistently higher at site 1 compared with site 2; again this systematic difference was not fully compensated by normalising against the direct-to-MGIT inoculum controls, as shown by the Bland-Altman plot (mean bias = −0.41 log_10_ CFU, Fig. 4d). However, the difference between the highest and lowest responses was consistent between runs (0.26 and 0.33 log_10_ CFU respectively), and all samples were within the 95% limits of agreement, which extended from −0.61 (95% CI, −0.89 to −0.54) to −0.21 (95% CI, −0.27 to 0.07) log_10_ CFU. Between sites 1 and 3, the median CV was 3.17% (range 0.39–8.62%) with an ICC of 0.83 (‘almost perfect’ agreement; Table 2). There was a small mean bias of 0.11 log_10_ CFU between sites 1 and 3 following normalisation but this was not significant; the Bland-Altman 95% limits of agreement extended from −0.26 (95% CI, −0.70 to −0.15) to 0.49 (95% CI, 0.37–0.93) log_10_ CFU and again all samples fell within this range (Fig. 4f).

**Table 2:** NHP MGIA inter-site reproducibility.

	Site 1 vs. Site 2 (*n* = 8)	Site 1 vs. Site 3 (*n* = 8)
CV (%)	14.19	3.17
ICC	0.57	0.83

The median CV and ICC is shown for shared sample sets assayed at different laboratory sites.

### Mycobacterial growth inhibition in the direct PBMC MGIA is enhanced following alternative routes of BCG administration and correlates with measures of protection from in vivo *M.tb* challenge at an individual animal level

**Fig. 5:**
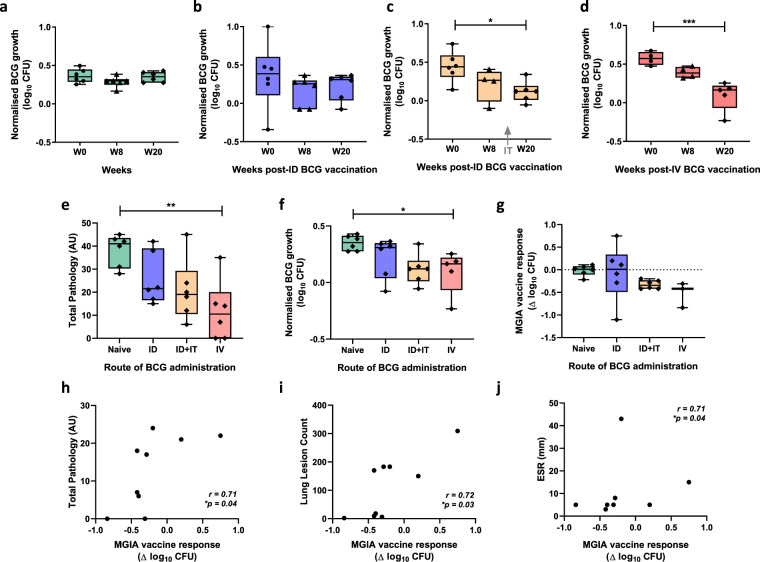
Mycobacterial growth inhibition is enhanced following alternative routes of BCG administration. Samples were collected from 24 Rhesus macaques from Study 4. Six animals were unvaccinated (green boxes), six received BCG vaccination by the intradermal (ID) route (blue boxes), six received BCG vaccination by the ID followed by intratracheal (IT) routes (orange boxes), and six received BCG vaccination by the intravenous (IV) route (red boxes). The MGIA (3 × 10^6^ cells, 500 CFU BCG in static 48-well plates) was performed using PBMC and autologous serum collected at baseline (circles), and at 8 weeks (triangles) and 20 weeks (diamonds) post-BCG vaccination in the (**a**) naïve, (**b**) ID BCG, (**c**) ID + IT BCG and (**d**) IV BCG vaccinated groups. At 21 weeks after primary vaccination, all animals were challenged by exposure to aerosol *M.tb* and total pathology was determined 2 weeks later (**e**). BCG growth in the MGIA (**f**) and MGIA vaccine response (post-vaccination growth – baseline growth) (**g**) at 20 weeks post-vaccination were compared between groups. Correlations are shown between the MGIA vaccine response (post-vaccination growth – baseline growth) at 20 weeks and (**h**) total pathology, (**i**) lung lesion count and (**j**) ESR using Spearman’s rank correlation coefficient. Points represent individual animals. Normalised mycobacterial growth is equal to (log_10_ CFU of sample – log_10_ CFU of inoculum control). Boxes indicate the median value with the interquartile range and whiskers indicate minimum and maximum values. A one-way ANOVA was performed with Dunnett’s multiple comparisons test (**a**–**d**) or Tukey’s multiple comparisons test (**e–g**) where *p-value < 0.05, **p-value < 0.01 and ***p-value < 0.001.

Samples were taken from 24 Rhesus macaques enrolled into Study 4. Six animals were unvaccinated controls, six received BCG vaccination by the intradermal (ID) route, six received BCG vaccination by the ID followed by intratracheal (IT) routes, and six received BCG vaccination by the intravenous (IV) route^35^. The optimised, standardised direct PBMC MGIA protocol was applied to samples taken at baseline, week 8 and week 20 (the closest time-point to day-of-challenge). Differences in MGIA control of BCG growth were not observed in the naïve or BCG ID groups over time, although there was a trend towards reduced growth at 8 weeks following ID BCG vaccination (Fig. 5a, b). However, there was a significant reduction in BCG growth at 20 weeks post-ID + IT BCG vaccination (*p* = 0.015, MD = 0.33 log_10_ CFU, 95% CI 0.07–0.59, one-way ANOVA with Dunnett’s post-test, *p* = 0.022, *F*(2, 13) = 5.18; Fig. 5c) and at 20 weeks post-IV BCG vaccination (*p* = 0.0007, MD = 0.48 log_10_ CFU, 95% CI 0.25–0.71; one-way ANOVA with Dunnett’s post-test, *p* = 0.001, *F*(2, 10) = 14.42; Fig. 5d) compared to baseline. There was also a reduction at 8 weeks following IV BCG vaccination (*p* = 0.04, *t*(3) = 3.60, MD = −0.18 log_10_ CFU, 95% CI −0.34 to −0.02, paired *t*-test), but this was no longer significant following correction for multiple comparisons.

All animals were challenged by exposure to aerosol *M.tb* at 21 weeks after primary vaccination. Clinical parameters measured at necropsy were time of disease control, lung pathology, total pathology, lung lesion count, lesion:lung ratio, % weight loss, ESR, and X-ray score. As previously reported, disease pathology was reduced, and disease control improved, by all BCG vaccination strategies compared with naïve animals. IV vaccination induced protection which surpassed that achieved by all other routes^35^. Total pathology score at necropsy was lower in all BCG vaccinated groups compared with naïve animals, which remained significant in the IV BCG vaccinated group following correction for multiple comparisons (*p* = 0.004, MD = 26.33 AU, 95% CI 7.76-44.9; one-way ANOVA with Tukey’s post-test, *p* = 0.0065, *F*(3, 20) = 5.48; Fig. 5e). BCG growth in the MGIA at 20 weeks was significantly lower in the ID + IT and IV BCG vaccinated groups compared with the naïve group using an unpaired *t*-test (*p* = 0.003, *t*(10) = 3.81, MD = −0.23 log_10_ CFU, 95% −0.37 to −0.10; and *p* = 0.013, *t*(9) = 3.07, MD = −0.25 log_10_ CFU, 95% CI −0.44 to −0.07 respectively), which remained significant in the IV vaccinated group following correction for multiple comparisons (*p* = 0.047, MD = 0.25 log_10_ CFU, 95% CI 0.002–0.51, one-way ANOVA with Tukey’s post-test, *p* = 0.034, *F*(3, 19) = 3.54; Fig. 5f).

MGIA vaccine response (post-vaccination growth – baseline growth) at the peak of the MGIA response (week 20) was significantly greater in the ID + IT and IV BCG vaccinated groups compared with the naïve group using an unpaired *t*-test (*p* = 0.0005, *t*(10) = 5.03, MD = −0.31 Δ log_10_ CFU, 95% CI −0.45 to −0.17; and *p* = 0.005, *t*(7) = 4.00, MD = −0.51 Δ log_10_ CFU, 95% CI −0.81 to −0.21 respectively), but this was not significant following correction for multiple comparisons (Fig. 5g). There was no association between MGIA outcome following BCG vaccination and any of the measures of in vivo protection on a per-individual basis when considering all animals. However, for some animals disease reached humane endpoint criteria necessitating euthanasia prior to the end of the study; when these animals were excluded from the analysis (ID BCG *n* = 3, ID + IT BCG *n* = 2 and IV BCG *n* = 1), there was a weak but significant association between MGIA vaccine response (post-vaccination growth – baseline growth) at the peak of response (20 weeks) following BCG vaccination and total pathology, lung lesion count and ESR (*p* = 0.02, *r* = 0.71; *p* = 0.02, *r* = 0.72; and *p* = 0.03, *r* = 0.71 respectively, Spearman’s, Fig. 5h–j).

## Discussion

We aimed to adapt the direct MGIA, previously described in humans and mice^20,21,39,42,43^, as a surrogate read-out of vaccine-induced protection against TB in the macaque model. The enhanced control of mycobacterial growth observed in the direct whole blood MGIA following BCG vaccination in cynomolgus macaques is consistent with in vivo data demonstrating a partially protective effect of BCG vaccination in this species^44,45^, and with our previous observations in rhesus macaques^29^ and humans^39^. Notably, the peak of the human MGIA response was reported at 8 weeks post-BCG vaccination with a reduced effect by 24 weeks; a kinetic mirrored in our study^39^. This may be related to immunogenicity following ID BCG vaccination, with a peak PPD-specific IFN-γ T-cell response at 4–8 weeks which wanes by 20–24 weeks^39,46^. A similar MGIA kinetic and correlation was observed for BCG or *M.tb* inocula, supporting the use of BCG as a surrogate in line with previous work^28,40^. While clumping issues with our initial BCG Pasteur stock and lower growth may have reduced intra-assay reproducibility compared with *M.tb*, the MGIA vaccine response (post-vaccination growth – baseline growth) was greater using BCG. On balance we pursued assay development using a standardised, homogeneous BCG Pasteur stock to maximise logistical feasibility and transferability. One advantage of the direct MGIA is that manipulations (eg. reporter genes) are not required to evaluate different mycobacterial strains and isolates, but it should be noted that the optimal MOI may be strain-specific.

The finding that intra-assay repeatability and sensitivity to detect vaccine-induced control of mycobacterial growth in the PBMC MGIA is improved by increasing cell concentration or mycobacterial input is consistent with human and mouse direct MGIA work^21,43^, and illustrates the critical importance of MOI optimisation. We further improved these parameters by co-culturing in 48-well plates rather than rotating 2 ml tubes, again in line with findings using human and mouse cells^42,43^, and likely due to CO_2_ availability and lack of mechanical perturbation. Cell number is limiting in macaque studies due to permitted blood collection volumes; we therefore recommend a rational compromise of 3 million cells per co-culture. Mean cell viability at the end of the co-culture period under these conditions (72%) is comparable to that reported for the human direct PBMC MGIA (73%)^43^. One limitation of these early studies was the use of pooled human serum; for the final study (and going forward) autologous timepoint-matched macaque serum was included to capture serum component (eg. antibody) influences. An additional potential limitation is the relatively older age of the animals in Studies 1 and 2, which may be associated with a weaker immune response to vaccination^47^, however we did observe in vivo protection following mycobacterial challenge in BCG-vaccinated older animals as reflected in the MGIA.

Following side-by-side training with end-users by a ‘lead operator’ as recommended by Smith et al.^48^, repeatability and intermediate precision of the optimised assay were consistently <10% CV, and inter-site reproducibility <20% CV. Some lower reproducibility outcomes were likely influenced by the homogeneous sample set used^49^; indeed for the comparison between sites 1 and 3, agreement was higher using samples with a broader dynamic range (between-sample standard deviation = 0.34 compared with 0.08 for the comparison between sites 1 and 2). Based on consistency agreements, our reproducibility values are comparable to the human direct PBMC MGIA^43^ and well within the 50% limit of acceptable variation suggested for the measurement of a bacterial target in a cell-based assay^50^. This affords confidence in assay transferability to maximise both scientific and 3Rs impact. However, we did observe some systematic differences, likely driven by variation in mycobacterial inoculum input (a difference of 0.2 log_10_ CFU and 0.5 log_10_ CFU between assay runs at site 1 and in the site 1–2 comparison respectively), despite exchanging a common BCG stock and normalising growth values against direct-to-MGIT inoculum controls. Importantly the delta between the highest and lowest values, which may be the most relevant measure, was consistent. Nonetheless, we recommend batching samples for direct comparisons, and further work should aim to standardise the mycobacterial inoculum and define an internal reference standard.

Sharpe et al.^35^ reported that IV BCG-induced protection against aerosol *M.tb* challenge surpassed that achieved by all other routes tested. Using samples from this study we replicated this finding using the direct PBMC MGIA, although sensitivity was lower than in vivo challenge. This is perhaps unsurprising, given the MGIA attempts to model a complex and dynamic biological system using cells present at a specific moment in time. Interestingly while growth inhibition in the ID BCG group was waning by week 20, consistent with Study 1 MGIA data presented here and with previous MGIA findings in humans^39^, this was not the case in the ID + IT and IV BCG groups, suggesting that protection afforded by these routes may be more durable. Indeed, immunogenicity measures remained higher for longer in the ID + IT and IV BCG groups and were significantly greater at day-of-challenge than in the ID BCG group^35^. This is also consistent with recent work demonstrating high levels of protection at 24 weeks post-IV (but not -ID) BCG vaccination in rhesus macaques^51^. Both studies observed higher specific T-cell responses in the periphery following IV BCG vaccination, which may drive the superior MGIA control observed^35,51^. However, Darrah et al. also noted a high frequency of antigen-responsive T-cells in the bronchoalveolar lavage, supporting the need for a complementary lung-based direct MGIA as recently described in the murine model^22^.

An NHP MGIA offers the unique opportunity for biological validation at a per-individual as well as per-group level. In Study 1, there was a significant correlation between post-challenge LN CFU recovery and *M.tb* growth in the whole blood MGIA at the peak response, and moreso the MGIA vaccine response (post-vaccination growth – baseline growth). While there was no correlation between MGIA outcome and biopsy CFU recovery, the value of this measure in macaques is unclear due to local tissue structure; BCG could not be detected in 5/7 naïve animals and biopsy CFU did not differ between naïve and BCG-vaccinated animals following high-dose BCG challenge^38^. These limitations may also account for the lack of correlation between individual challenge outcome and MGIA in Study 2, where biopsy CFU was the only in vivo read-out. In addition to Study 1, MGIA vaccine response (post-vaccination growth–baseline growth) correlated with multiple post *M.tb*-challenge protection measures in Study 3 and, to a lesser extent, Study 4. Taken together, this suggests that post-vaccination control of growth relative to baseline is a better surrogate of in vivo protection than absolute inhibition at a given time-point. One limitation of these *M.tb* challenge studies is that pulmonary *M.tb* burden was not measured in Study 4, and did not correlate with MGIA outcome in Study 3, although this endpoint has not been standardised^8^.

A degree of biological validation was achieved using both cynomolgus and rhesus macaques of different genetic backgrounds. While rhesus macaques are typically more susceptible to *M.tb* infection and disease than cynomolgus, the Mauritian cynomolgus genotype used here more closely resembles the susceptibility profile of rhesus^8,30^. BCG vaccine efficacy has also been shown to be superior in cynomolgus compared with rhesus macaques^52^, and indeed we observed strongly enhanced MGIA control post-BCG vaccination in this species. The direct NHP MGIA is now being implemented for further evaluation at the end-user institutes, where improved mycobacterial growth inhibition has been demonstrated following *M.tb* infection^30^, consistent with studies in recently *M.tb*-infected humans^27,28^. Additional work should aim to further improve assay sensitivity, evaluate performance in trials of novel TB vaccine candidates and interrogate immune mechanisms of control. In the human direct MGIA, mycobacterial growth inhibition has been variously associated with ratio of monocytes to lymphocytes^27,28,53^, polyfunctional CD4 ^+^ T-cells^54^, B-cell and IgG1 responses^28^, cytokines associated with trained immunity^27^ and distinct transcriptomic profiles^55^; similar mechanisms may contribute in the macaque^56^.

In conclusion, we have indicated predictive utility of the NHP MGIA across different macaque species and challenge contexts. Our findings support the potential of the direct NHP MGIA as a promising tool to facilitate the early testing of novel TB vaccine candidates and reduce those progressing to virulent *M.tb* challenge studies. Application of the assay to assess control of different mycobacterial strains and isolates, and immune mechanisms involved, would further reduce the number of in vivo challenge experiments in macaques. Importantly, biologically validating the MGIA as a reliable surrogate of vaccine-induced protection (against direct in vivo measures such as lung pathology and/or bacterial burden) in the NHP model, would then allow it to be applied to human studies in which it is not possible to assess such measures.

## Methods

### Macaque studies

To minimise the number of animals used in line with the 3Rs principles, samples were taken from ongoing or historical vaccination studies for which sample sizes were calculated based on power to observe a difference between groups in protection from in vivo challenge. The animals from which samples were used in MGIA studies were cynomolgus macaques of Mauritian genotype (Study 1, *n* = 8), rhesus macaques of Indian genotype (Study 2, *n* = 12 and Study 4, *n* = 24) or rhesus macaques of Chinese genotype (Study 3, *n* = 24). In all cases, animals were captive-bred for research purposes, and a single animal was considered an experimental unit; samples collected from a total of 67 animals were used. The PBMC and serum samples used for MGIA analysis were not selected but were provided according to availability, and were unblinded with individual animal identifiers provided. The samples represent a spectrum of more or less protected phenotypes for correlative investigation against possible MGIA signals. Laboratory staff were blinded as to levels of in vivo protection when samples were tested in the MGIA. A summary of the samples used for MGIA assays, the studies from which they originated, animal characteristics and treatment group comparisons is provided in Table 3.

**Table 3:** Summary of samples used in MGIA studies.

Study	Species	Groups (*n*)	BCG vaccination	In vivo challenge	Refs.
1	Cynomolgus, Mauritian Female 14.3–15.6 years	BCG ID (8)	BCG Danish (SSI) 2–8 × 10^5^ CFU	BCG Danish (SSI) 1–4 × 10^6^ CFU ID route 21 weeks post-BCG	^38^
2	Rhesus, Indian Female 14.3–15.6 years	Naïve controls (6) BCG ID (6)	BCG Danish (SSI) 2–8 × 10^5^ CFU	BCG Danish (SSI) 2–8 × 10^5^ CFU ID route 21 weeks post-BCG	^38^
3	Rhesus, Chinese Male 2.5–3.4 years	Naïve controls (9) BCG ID (15)	BCG Danish (SSI) 2–8 × 10^5^ CFU	M.tb Erdman strain K01 (Aeras) 500 CFU Endobronchial instillation route 38 weeks post-BCG	n/a
4	Rhesus, Indian Male 2.1–2.8 years	Naïve controls (6) BCG ID (6) BCG ID + IT (6) BCG IV (6)	BCG Danish (SSI) 2–8 × 10^5^ CFU ID 2–8 × 10^5^ CFU ID + 2–8 × 10^6^ CFU IT 2–8 × 10^6^ CFU IV	M.tb Erdman strain K01 (BEI) 100 CFU Aerosol route 21 weeks post-BCG	^35^

The following samples do not have MGIA data because either PBMC and/or timepoint-matched autologous serum were unavailable, because an insufficient number of viable cells were recovered post-thawing, or occasionally because MGIA cultures were contaminated (TTP < 48 h): one animal in the BCG vaccinated group from Study 2 for the MGIA with 5 × 10^6^ cells with 500 CFU BCG (Fig. 2b); one animal in the Study 3 naïve group at 8 weeks for the 3 × 10^6^ cells in-tube assay (Fig. 3b), one animal in the BCG vaccinated group at baseline for both in-plate assays (Fig. 3c, d) and one animal in the BCG vaccination group at 8 weeks for the 3 × 10^6^ cells in-plate assay (Fig. 3d); two animals in the Study 4 ID + IT group at week 8 (Fig. 5c), two animals from the IV group at weeks 0 and 8 and 1 animal from the IV group at week 20 (Fig. 5d); one animal from the site 1 and 2 comparison at site 1 (Fig. 4c).

For the original in vivo studies, animals were obtained from established breeding colonies at Public Health England in the UK (Studies 1, 2 and 4), or purchased by the Biomedical Primate Research Centre in 2008 from a certified provider in the Netherlands (Study 3). None of the animals had been used previously for experimental procedures. Animals were socially housed throughout the experiments and provided with enrichment in the form of food and non-food items on a daily basis; animal welfare was monitored daily. In all studies, absence of previous exposure to mycobacterial antigens was confirmed by a tuberculin skin test applied as part of colony management practice and/or by screening using an ex vivo IFN-γ ELISpot upon mycobacterial recall stimulation of PBMC with purified protein derivative (PPD) of *M.tb* (Mabtech, Sweden; U-Cy Tech, Utrecht). Animals were sedated by intramuscular (IM) injection of ketamine hydrochloride (Ketaset, 100 mg/ml, Fort Dodge Animal Health Ltd, Southampton, UK; 10 mg/kg) for procedures requiring removal from their housing.

The macaque studies included in this paper complied with all relevant ethical regulations for animal testing and research. For Studies 1, 2 and 4, study design and procedures were approved by the Public Health England Porton Down Animal Welfare and Ethical Review Committee and authorized under an appropriate UK Home Office project license. Animals were housed in compatible social groups in accordance with the Home Office (UK) Code of Practice for the Housing and Care of Animals Used in Scientific Procedures (1989) and the National Centre for Refinement, Reduction and Replacement (NC3Rs) Guidelines on Primate Accommodation, Care and Use, August 2006 (NC3Rs, 2006). For Study 3, ethical approval was obtained from the independent animal ethics committee (Dierexperimentencommissie, DEC, Netherlands; dossier number 579). Housing and animal care procedures were in compliance with Dutch law on animal experiments, European directive 86/609/EEC, and the ‘Standard for Humane Care and Use of Laboratory Animals by Foreign Institutions’ provided by the Department of Health and Human Services of the United States National Institutes of Health (NIH, identification number A5539-01). The BPRC is accredited by the American Association for Accreditation of Laboratory Animal Care since 2012.

In Studies 1, 2 and 4, treatment groups were randomly assigned to socially compatible cohorts (single gender, behaviourally harmonious groups) using software-generated random number allocations (Microsoft Excel). In Study 3, animals were stratified into groups on the basis of age, body weight and indicators for social housing; specific treatment was assigned to groups randomly with treatment groups randomly housed across animal rooms throughout classified experimental facilities at BPRC.

In Studies 1, 2 and 3, *n* = 8, *n* = 6 and *n* = 15 animals respectively received an adult human dose of BCG Danish strain 1331 (SSI, Copenhagen) 2–8 × 10^5^ CFU intradermally (ID) into the upper arm under sedation. The BCG vaccine was prepared and administered according to the manufacturer’s instructions for preparation of vaccine for administration to human adults, by addition of 1 ml Sauton’s diluent to a lyophilised vial. In Study 4, animals were randomised to be unvaccinated controls (*n* = 6), to receive 2–8 × 10^5^ CFU BCG ID (*n* = 6), to receive 2–8 × 10^6^ CFU intravenously (IV) into the femoral vein of the left leg (*n* = 6) or to receive 2–8 × 10^5^ CFU BCG ID followed by a second vaccination with 2–8 × 10^6^ CFU BCG intratracheally (IT; *n* = 6) 12 weeks later. For the IT boost, 1 ml BCG was delivered using an endotracheal catheter gauge 8 FG inserted into the lung to a depth of 15 cm, with the catheter flushed through using 0.5 ml sterile PBS^35^.

At 21 weeks post-BCG vaccination, all animals in Studies 1 and 2 were challenged with 1–4 × 10^6^ CFU and 2–8 × 10^5^ CFU respectively of BCG Danish strain 1331 (SSI, Copenhagen) by the ID route into the upper arm. Two weeks after BCG challenge, a skin biopsy of the challenge site was taken under anaesthesia. The area of skin for biopsy was cleaned with 4% w/v chlorhexidine preparation (Hibiscrub, Regent Medical Overseas Ltd, UK) and 1–2 ml of local anaesthetic (lignocaine, 10 mg/ml with adrenaline, 5 µg/ml, Xylocaine, AstraZeneca UK) injected subcutaneously in and around the BCG infection site. After 1–2 mins, a 4-mm biopsy was collected using a disposable biopsy punch (William Needham & Associates, UK), and the sample was snap frozen. A gauze swab was applied with pressure to the skin for 0.5–1 min and the site cleaned with a moist swab^38^. In Study 1, animals were then euthanised under anaesthesia by intra-cardiac injection of a lethal dose of pentobarbitone sodium (Dolelethal, Vétoquinol UK Ltd., 140 mg/kg). A full necropsy was performed immediately, gross pathology assessed and the left and right axillary lymph nodes were collected and snap frozen^38^. Outcome measures were mycobacterial burden recovered from skin biopsy (Studies 1 and 2) and lymph nodes (Study 1), quantified by culture on solid agar and qPCR, the results of which have been reported elsewhere^38^.

In Study 3, animals were challenged by endobronchial instillation of 500 CFU of *M.tb* Erdman strain K01 at 38 weeks after BCG vaccination. Infectious challenge was performed in two sessions within 2–3 h from preparing the inoculum from frozen *M.tb* stock, treating animals from different groups in a random order. For the endobronchial instillation procedure, ketamine (5 mg kg^−1^) was supplemented with intramuscular medetomidine (0.04 mg kg^−1^) and an analgesic sprayed into the larynx. The condition of the animals was monitored by daily observation throughout the study and recorded, with particular attention to behaviour, stool, appetite and respiratory labour. At the end of the study (50 weeks post-challenge), or when a humane endpoint was reached to limit possible discomfort, animals were euthanized by intravenous injection of pentobarbital under ketamine sedation. Humane endpoint was called by experienced veterinary staff upon occurrence of adverse indicators including depression or withdrawn behaviour, abnormal respiration (dyspnoea) or loss of 20% of peak post-challenge weight. All veterinary staff members were blinded to the group allocation of individual animals. Outcome measures were time to humane endpoint (if the fixed endpoint was not reached), total pathology, lung pathology, extra-thoracic pathology, lung CFU, C-reactive protein (CRP) and weight change.

In study 4, animals were challenged by exposure to a target dose of 100 CFU of aerosolised *M.tb* Erdman strain K01 (BEI resources) delivered into the lungs at 21 weeks after primary vaccination. Mono-dispersed bacteria in particles of mean diameter 2 µm were generated using a 3-jet Collision nebuliser (BGI Incorporated, MA, USA) and delivered to the nares of sedated animals placed in a ‘head-out’ plethysmography chamber (Buxco, Wilmington, NC, USA) via a veterinary anaesthesia mask (modified to permit the flow of aerosol over the nose) in conjunction with a modified Henderson apparatus. This allowed aerosol to be delivered simultaneously with the measurement of respiration rate and volume. One animal from each group was exposed in sequence to minimise potential confounders in order of treatment. Animals were monitored daily for behavioural abnormalities including depression, aggression, withdrawal, changes in feeding pattern, altered respiration rate and coughing. Animals were weighed, had rectal temperature measured and were examined for gross abnormalities whenever procedures (vaccination, blood sample collection, mycobacterial challenge or euthanasia) were conducted. Haemoglobin levels were measured using a HaemaCue haemoglobinometer (Haemacue Ltd, Dronfield, UK) and erythrocyte sedimentation rates (ESR) were measured using the Sediplast system (Guest Medical, Edenbridge, UK). Humane endpoints were determined by experienced primatology staff and based on a predetermined combination of adverse indicators: depression or withdrawn behaviour, abnormal respiration (dyspnoea), loss of 20% of peak post-challenge weight, ESR levels elevated above normal (>20 mm), haemoglobin level below normal limits (<100 g/dl), increased temperature (>41 °C) and severely altered thoracic radiograph^35^. Outcome measures were time of disease control, lung pathology, total pathology, lung lesion count, lesion:lung ratio, % weight loss, ESR and X-ray score, the results of which have been reported elsewhere^35^.

### Mycobacterial growth inhibition assay

In-house BCG Pasteur and *M.tb* H37Rv stocks were grown in Middlebrook 7H9 medium with 10% oleic acid dextrose catalase (OADC) to mid-log phase, divided into 1 ml aliquots and stored at −80 °C until required. Aliquots were thawed at room temperature immediately prior to inoculation and diluted to the titres specified in RPMI (containing 2 mM l-glutamine and 25 mM HEPES). For harmonisation experiments, frozen aliquots of a single batch of BCG Pasteur stock (Aeras, Rockville, USA) grown in Middlebrook 7H9 medium with 10% OADC and 0.05% tyloxapol were circulated and serially diluted prior to inoculation in a standardised way between sites. The BCG Pasteur stock prepared by Aeras and tested for viability and reproducibility is available for future inter-site standardisation. The whole blood MGIA was performed using fresh whole blood collected in heparin tubes. For the PBMC MGIA, cryopreserved PBMC were rapidly thawed in a water bath at 37 °C until a small amount of frozen material remained. Samples were gradually added to 10 ml RPMI (containing 10% foetal calf serum and 2 mM l-glutamine) using a Pasteur pipette. The cryovial was rinsed using 1 ml of fresh medium and added to the corresponding tube, which was then centrifuged at 350×*g* for 7 min. Supernatants were removed by inversion and cells resuspended at an approximate concentration of 2–3 × 10^6^ cells per ml of RPMI (containing 10% foetal calf serum and 2 mM l-glutamine) and 2 µl/ml of 25 U benzonase added to each tube. Cells were rested at 37 °C for 2 h with 5% CO_2_ before counting.

The direct whole blood MGIA was adapted from the methods of Wallis et al.^57^. Duplicate 2 ml screw-cap tubes containing 300 μl of fresh whole blood were mixed with 300 μl RPMI (containing 2 mM l-glutamine, 25 mM HEPES and 10% pooled human AB serum) seeded with ~500 CFU of BCG Pasteur or *M.tb* H37Rv. For the direct PBMC ‘in-tube’ assay, 600 µl RPMI (containing 2 mM l-glutamine and 25 mM HEPES and 10% pooled human AB serum) seeded with 1 × 10^6^ PBMC and ~500 CFU BCG Pasteur (unless otherwise specified) was added to duplicate 2 ml screw-cap tubes. The co-cultures were incubated on a 360° rotator at 37 °C for 96 h, after which time tubes were microcentrifuged at 15,300×*g* for 5 mins (whole blood) or 10 mins (PBMC) and the supernatant carefully removed by pipetting. Cells were lysed with the addition of 1 ml (whole blood) or 500 µl (PBMC) sterile water and the tubes pulse-vortexed at 0, 5 and 10 mins. For the PBMC assay, the 500 µl of lysate was then transferred directly to a BACTEC MGIT tube supplemented with PANTA antibiotics (polymyxin B, amphotericin B, nalidixic acid, trimethoprim and azlocillin) and OADC enrichment broth (Becton Dickinson, UK). For the whole blood assay, lysates were microcentrifuged at 15,300×*g* for 10 mins and the supernatant carefully removed by pipetting. Pellets were resuspended in 500 µl of BACTEC MGIT medium (supplemented with PANTA and OADC), pulse vortexed and added to BACTEC MGIT tubes (supplemented with PANTA and OADC).

For the direct PBMC ‘in-plate’ MGIA, 3 × 10^6^ PBMC and ~500 CFU BCG Pasteur (unless otherwise specified) in a total volume of 480 μl RPMI (containing 2mM l-glutamine and 25 mM HEPES), plus 120 μl autologous serum matched to animal and time-point (or pooled human AB serum in studies where this was not available) were added per well of a 48-well plate (total volume 600 μl per well). Co-cultures were incubated at 37 °C for 96 h and then transferred to 2 ml screw-cap tubes and centrifuged at 15,300×*g* for 10 min. During this time, 500 μl sterile water was added to each well to lyse adherent monocytes and release intracellular mycobacteria. Supernatants were carefully removed from the 2 ml screw-cap tubes by pipetting, and water from the corresponding well added to the remaining pellet. Tubes were pulse vortexed and the lysate transferred to BACTEC MGIT tubes supplemented with PANTA antibiotics and OADC (Becton Dickinson, UK).

At the end of all MGIA protocols described, MGIT tubes were placed on the BACTEC 960 machine (Becton Dickinson, UK) and incubated at 37 °C until the detection of positivity by fluorescence. On day 0, duplicate direct-to-MGIT inoculum control tubes were set up by inoculating supplemented BACTEC MGIT tubes with the same amount of mycobacteria as the samples. The time to positivity (TTP) read-out was converted to log_10_ CFU using stock standard curves of TTP against inoculum volume and CFU. Results are presented as ‘normalised mycobacterial growth’ which is equal to (log_10_ CFU of sample – log_10_ CFU of inoculum control) to correct for variation in inoculum between mycobacterial stocks, batches and aliquots. ‘Vaccine response’ following BCG vaccination was calculated as (post-vaccination normalised growth – baseline normalised growth), and is presented as Δlog_10_ CFU.

### Statistical analysis

Statistical analysis was performed using GraphPad Prism v.7 and IBM SPSS v.25. Normality of data was determined using a Shapiro-Wilk test. For parametric data with multiple groups, a one-way ANOVA or repeated-measures ANOVA (for time-course data) was conducted followed by a Tukey’s post-test (comparison of all groups) or Dunnett’s/Sidak’s multiple comparisons test (comparison of preselected pairs of groups ie. post-vaccination timepoints vs. baseline). For comparisons between two groups of normally-distributed data, a two-sided *t*-test or paired *t*-test was used. For comparisons between two groups of non-parametric data or small sample sizes, a two-sided Mann Whitney or Wilcoxon matched-pairs signed rank test was conducted. Effect sizes are estimated as the difference between the means (MD) of the two groups. A two-tailed Spearman’s rank correlation was used to determine associations between two measures. For determination of intra-assay repeatability, intermediate (inter-assay) precision and inter-site reproducibility, the coefficients of variation (CV) (standard deviation/mean × 100) and intra-class correlation coefficients (ICC) (two-way mixed model, consistency agreement, single measures) were calculated using raw time-to-positivity (TTP) values. ICC categories for interpreting kappa values were taken from the guidelines of Landis and Koch^58^. Following confirmation of normality in the distribution of differences between paired measurements, the Bland-Altman method was used to analyse the intermediate precision and inter-site reproducibility of normalised mycobacterial growth values^59^. 95% confidence intervals for the Bland-Altman limits of agreement were calculated using the methods described by Carkeet^60^.

### Reporting summary

Further information on research design is available in the Nature Research Reporting Summary linked to this article.

## Supplementary information

Supplementary Information

Reporting Summary

## Data Availability

The datasets generated and analysed during the current study are available from the corresponding author on reasonable request.
