# Supplementary material for: A non-human primate in vitro functional assay for the early evaluation of TB vaccine candidates
Source: NPJ Vaccines. 2021 Jan 4;6:3. doi: 10.1038/s41541-020-00263-7 (PMC7782578; doi:10.1038/s41541-020-00263-7)
Supplement: Supplementary file 1 — Supplementary Information [file 41541_2020_263_MOESM1_ESM.pdf]

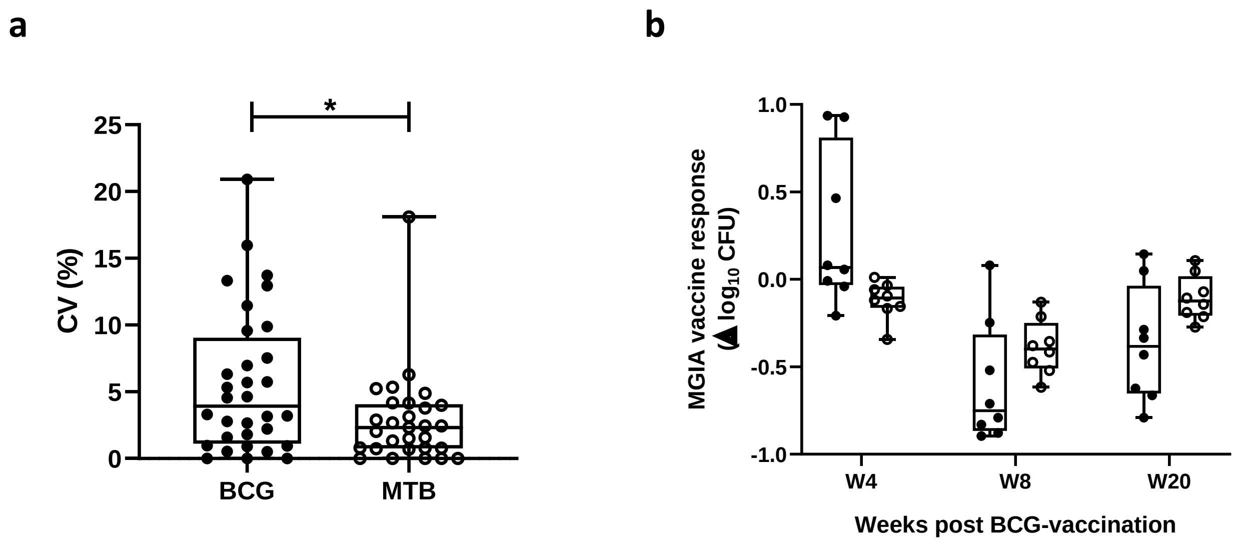

Supplementary Figure 1. Comparison of using BCG or *M.tb* as the MGIA inoculum. Samples were collected from 8 cynomolgus macaques from Study 1 that received BCG vaccination by the ID route. The MGIA was performed using whole blood taken at baseline, 4 weeks, 8 weeks and 20 weeks post-BCG vaccination using BCG Pasteur (closed circles) or *M.tb* H37Rv (open circles) as the inoculum. The coefficient of variation (CV) was measured between replicate co-cultures and compared between inocula using a Wilcoxon test (a) where * indicates a p-value of <0.05. The MGIA vaccine response (post-vaccination growth – baseline growth) was compared between inocula at each post-vaccination time-point (b). Points represent individual animals with the mean of two co-culture replicates. Boxes indicate the median value with the interquartile range and whiskers indicate the minimum and maximum values.

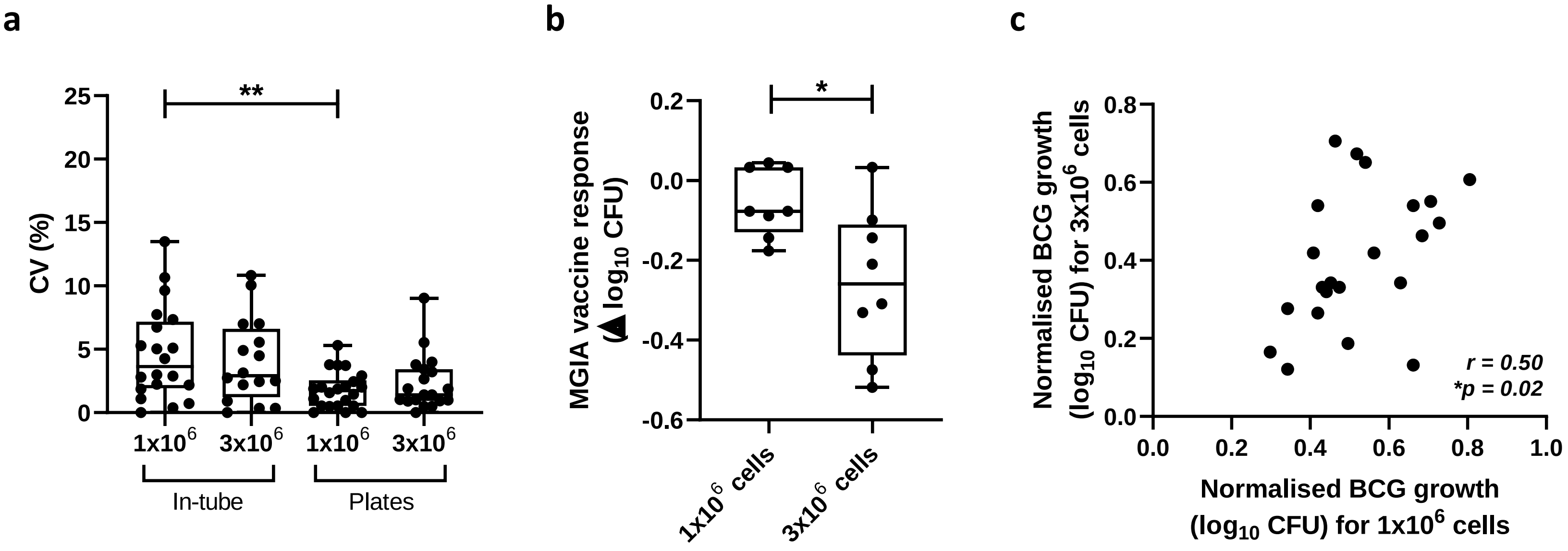

Supplementary Figure 2. Optimisation of the NHP direct PBMC MGIA. Samples were collected from 24 Rhesus macaques from Study 3, 9 of which were naïve unvaccinated controls and 15 of which received BCG vaccination by the intradermal (ID) route. The MGIA was performed using PBMC from baseline and 8 weeks post-BCG vaccination according to the original 'in-tube protocol' co-culturing in 2ml screw-cap rotating tubes or in static 48-well tissue culture plates. Co-cultures contained either $1x10^6$ PBMC or $3x10^6$ PBMC. The coefficient of variation (CV) was measured between replicate co-cultures and compared between assay conditions using a one-way ANOVA with Tukey's multiple comparisons test (a). The MGIA vaccine response (post-vaccination growth – baseline growth) was compared between using $1x10^6$ PBMC or $3x10^6$ PBMC in 48-well tissue culture plates using a paired t-test (b), and the association between normalised BCG growth in the MGIA using $1x10^6$ PBMC or $3x10^6$ PBMC was tested using Spearman's rank correlation (c). Boxes indicate the median value with the interquartile range and whiskers indicate the minimum and maximum values. * indicates a p-value of <0.05 and ** indicates a p-value of <0.01.
