# Supplementary material for: A non-human primate in vitro functional assay for the early evaluation of TB vaccine candidates
Source: NPJ Vaccines. 2021 Jan 4;6:3. doi: 10.1038/s41541-020-00263-7 (PMC7782578; doi:10.1038/s41541-020-00263-7)
Supplement: Supplementary file 2 — Reporting Summary [file 41541_2020_263_MOESM2_ESM.pdf]

nature research

Corresponding author(s): Rachel Tanner

Last updated by author(s): Nov 17, 2020

Reporting Summary

Nature Research wishes to improve the reproducibility of the work that we publish. This form provides structure for consistency and transparency in reporting. For further information on Nature Research policies, see our Editorial Policies and the Editorial Policy Checklist.

Statistics

For all statistical analyses, confirm that the following items are present in the figure legend, table legend, main text, or Methods section.

n/a	Confirmed	
☐	☒	The exact sample size (*n*) for each experimental group/condition, given as a discrete number and unit of measurement
☐	☒	A statement on whether measurements were taken from distinct samples or whether the same sample was measured repeatedly
☐	☒	The statistical test(s) used AND whether they are one- or two-sided<br>*Only common tests should be described solely by name; describe more complex techniques in the Methods section.*
☐	☒	A description of all covariates tested
☐	☒	A description of any assumptions or corrections, such as tests of normality and adjustment for multiple comparisons
☐	☒	A full description of the statistical parameters including central tendency (e.g. means) or other basic estimates (e.g. regression coefficient) AND variation (e.g. standard deviation) or associated estimates of uncertainty (e.g. confidence intervals)
☐	☒	For null hypothesis testing, the test statistic (e.g. F, t, r) with confidence intervals, effect sizes, degrees of freedom and P value noted<br>*Give P values as exact values whenever suitable.*
☒	☐	For Bayesian analysis, information on the choice of priors and Markov chain Monte Carlo settings
☒	☐	For hierarchical and complex designs, identification of the appropriate level for tests and full reporting of outcomes
☐	☒	Estimates of effect sizes (e.g. Cohen's d, Pearson's r), indicating how they were calculated

Our web collection on statistics for biologists contains articles on many of the points above.

Software and code

Policy information about availability of computer code

Data collection	No software was used.
Data analysis	Statistical analysis was performed using GraphPad Prism v.7 and IBM SPSS v.25.

For manuscripts utilizing custom algorithms or software that are central to the research but not yet described in published literature, software must be made available to editors and reviewers. We strongly encourage code deposition in a community repository (e.g. GitHub). See the Nature Research guidelines for submitting code & software for further information.

Data

Policy information about availability of data

All manuscripts must include a data availability statement. This statement should provide the following information, where applicable:

- Accession codes, unique identifiers, or web links for publicly available datasets
- A list of figures that have associated raw data
- A description of any restrictions on data availability

The MGIA data sets generated and analyzed in this present study are available from the corresponding authors upon reasonable request. Likewise, upon request biomaterials that are still available from this study could be shared for further research.

Field-specific reporting

Please select the one below that is the best fit for your research. If you are not sure, read the appropriate sections before making your selection.

☒ Life sciences ☐ Behavioural & social sciences ☐ Ecological, evolutionary & environmental sciences

For a reference copy of the document with all sections, see nature.com/documents/nr-reporting-summary-flat.pdf

Life sciences study design

All studies must disclose on these points even when the disclosure is negative.

Sample size	Sample sizes for the original in vivo study designs were powered to observe differences in measures of protection from mycobacterial challenge, while minimising the number of non-human primates used in line with the 3Rs principles. The PBMC and serum samples used for MGIA analysis in this manuscript were not selected but were provided according to availability. The maximum number available from each study were used.
Data exclusions	As stated in the manuscript, the following samples do not have MGIA data because either PBMC and/or timepoint-matched autologous serum were unavailable, because an insufficient number of viable cells were recovered post-thawing, or occasionally because MGIA cultures were contaminated (TTP <48 hours): one animal in the BCG vaccinated group from Study 2 for the MGIA with 5x106 cells with 500 CFU BCG (Figure 2b); one animal in the Study 3 naïve group at 8 weeks for the 3x106 cells in-tube assay (Figure 3b), one animal in the BCG vaccinated group at baseline for both in-plate assays (Figure 3c-d), and one animal in the BCG vaccination group at 8 weeks for the 3x106 cells in-plate assay (Figure 3d); 2 animals in the Study 4 ID+IT group at week 8 (Figure 5c), 2 animals from the IV group at weeks 0 and 8, and 1 animal from the IV group at week 20 (Figure 5d); one animal from the site 1 and 2 comparison at site 1 (Figure 4c). or Study 4, some animals reached humane endpoint criteria necessitating euthanasia prior to the end of the study; the data was analysed both with and without these animals excluded and both outcomes are described in the manuscript.
Replication	While specific individual experiments could not be repeated due to limited sample availability, the finding of detecting a BCG-vaccine induced MGIA response was repeated across 4 independent macaque studies for validation. The finding of a correlation between in vitro MGIA outcome and in vivo protection from mycobacterial challenge was repeated in 3 independent macaque studies for validation. Section 3.3 of the paper is dedicated to assessing intra-assay, inter-assay and inter-site repeatability.
Randomization	In Studies 1, 2 and 4, treatment groups were randomly assigned to socially compatible cohorts (single gender, behaviourally harmonious groups) using software-generated random number allocations (Microsoft Excel). In Study 3, animals were stratified into groups on the basis of age, body weight and indicators for social housing; specific treatment was assigned to groups randomly with treatment groups randomly housed across animal rooms throughout classified experimental facilities at BPRC. Due to limited cell availability, paired samples from Study 3 collected at baseline and 8 weeks post-BCG vaccination were randomised to be tested using either the in-tube protocol with 1x106 and 3x106 cells (n=6 naïve and n=6 BCG vaccinated animals), or in a separate experiment, the 48-well plate protocol with 1x106 and 3x106 cells (n=3 naïve and n=9 BCG vaccinated animals). The animals randomised to the in-tube or plate MGIA conditions showed similar levels of in vivo protection by all primary outcomes.
Blinding	All veterinary staff members were blinded to the group allocation of individual animals. The PBMC and serum samples used for MGIA analysis were not selected but were provided according to availability, and were unblinded with individual animal identifiers provided; laboratory staff were blinded as to levels of in vivo protection when samples were tested in the MGIA.

Reporting for specific materials, systems and methods

We require information from authors about some types of materials, experimental systems and methods used in many studies. Here, indicate whether each material, system or method listed is relevant to your study. If you are not sure if a list item applies to your research, read the appropriate section before selecting a response.

Materials & experimental systems

n/a	Involved in the study	
☒	☐	Antibodies
☒	☐	Eukaryotic cell lines
☒	☐	Palaeontology and archaeology
☐	☒	Animals and other organisms
☒	☐	Human research participants
☒	☐	Clinical data
☒	☐	Dual use research of concern

Methods

n/a	Involved in the study	
☒	☐	ChIP-seq
☒	☐	Flow cytometry
☒	☐	MRI-based neuroimaging

Animals and other organisms

Policy information about studies involving animals; ARRIVE guidelines recommended for reporting animal research

Laboratory animals	Animals in Study 1 were cynomolgus Mauritian macaques, female, 14.3-15.6 years; animals in Study 2 were rhesus Indian macaques,

Laboratory animals	female, 14.3-15.6 years; animals in Study 3 were rhesus Chinese macaques, male, 2.5-3.4 years; animals in Study 4 were rhesus Indian macaques, male, 2.1-2.8 years.
Wild animals	The study did not involve wild animals.
Field-collected samples	The study did not involve samples collected from the field.
Ethics oversight	For Studies 1, 2 and 4, study design and procedures were approved by the Public Health England Porton Down Animal Welfare and Ethical Review Committee and authorized under an appropriate UK Home Office project license. For Study 3, ethical approval was obtained from the independent animal ethics committee (Dierexperimentencommissie, DEC, Netherlands; dossier number 579).

Note that full information on the approval of the study protocol must also be provided in the manuscript.
